# Supplementary material for: Complex pain phenotypes: Suicidal ideation and attempt through latent multimorbidity
Source: PLoS One. 2022 Apr 29;17(4):e0267844. doi: 10.1371/journal.pone.0267844 (PMC9053801; doi:10.1371/journal.pone.0267844)
Supplement: S1 Table — (DOCX) [file pone.0267844.s001.docx]

S1 Table. ICD-9-CM diagnostic code definitions among Post-9/11 Veterans.

| Condition | International Classification of Disease, 9^th^ Revision, Clinical Modification Codes (ICD-9-CM) |
| --- | --- |
| Back & neck pain | 720, 721.3, 721.4, 721.5, 721.6, 721.7, 721.8, 721.9, 722.1, 722.2, 722.3, 722.5, 722.70, 722.72, 722.73, 722.80, 722.82, 722.83, 722.90, 722.92, 722.93, 724, 847.4, 847.2, 847.3, 847.9, 805.2, 805.3, 805.4, 805.5, 805.6, 805.7, 805.8, 805.9, 839.5, 839.42, 737.1, 737.3, 738.4, 738.5, 739.2, 739.3, 739.4, 756.1; 721.0, 722.0, 722.71, 722.81, 722.91, 723, 805.0, 805.1, 839.0, 839.1, 847.0 |
| Other musculoskeletal pain | 715.92, 715.93, 719.42, 719.43, 719.44, 726.3, 726.4, 727.03, 727.04, 727.05, 727.06, 727.62, 727.63, 727.64, 719.41, 726.0, 726.1, 726.2, 727.61, 719.47, 726.7, 727.06, 727.1, 727.68, 728.71, 904.6, 715.15, 71516, 715.95, 715.98, 727.67, 726.6, 727.51, 726.71, 726.72, 727.65, 727.66, 727.67, 727.83, 729.2, 357, 353, 351.1, 352.1, 350, 337.2, 053, 356, 355, 354, 338.3, 307.80, 307.89, 338.0, 338.2, 338.4, 724.1, 729.1, 780.96, 524.6, 784.92, 379.91, 388.71, 388.72 |
| Headache | 339, 346, 784.0, 307.81 |
| Post-traumatic Stress Disorder | 309.81 |
| Depression | 296.2, 296.3, 311 |
| Anxiety | 300.0, 300.2, 300.3 |
| Substance Use Disorder | 291, 292, 303, 304, 305 (excluding 305.1) |
| Insomnia | 780.51, 780.52, 327.0, 307.4 |
| Attention impairment | 314, 79951 |
| Cognitive dysfunction | 290, 291.2, 046.1, 046.3, 294.0, 294.1,294.8, 294.9, 331.0, 331.1, 331.2, 331.3, 331.4, 331.5, 331.6, 331.7, 331.9, 331.81, 331.82, 331.89, 331.83 |
| Mild Traumatic Brain Injury | 800.00, 800.01, 800.02, 800.06, 800.09, 800.50, 800.51, 800.52, 801.00, 801.01, 801.02, 801.06, 801.09, 801.50, 801.51, 801.52, 803.00, 803.01, 803.02, 803.06, 803.09, 803.50, 803.51, 803.52, 804.00, 804.01, 804.02, 804.06, 804.09, 804.50, 804.51, 804.52, 850.0, 850.1, 850.11, 310.2, 959.01, 850, 850.5, 850.9 |
| Adverse Events |  |
| Suicidal ideation | V6284 |
| Suicide attempt | E950, E952, E953, E953, E954, E955, E956, E957, E958, E959 |
