# Supplementary material for: Complex pain phenotypes: Suicidal ideation and attempt through latent multimorbidity
Source: PLoS One. 2022 Apr 29;17(4):e0267844. doi: 10.1371/journal.pone.0267844 (PMC9053801; doi:10.1371/journal.pone.0267844)
Supplement: S2 Table — (DOCX) [file pone.0267844.s002.docx]

**S2 Table. Binomial logistic regression by complex pain phenotype for suicidal ideation or attempt.**

| **Characteristic** | **No adjustment** | **P value** | **Short set** | **P value** | **Long set** | **P value** |
| --- | --- | --- | --- | --- | --- | --- |
| **Complex pain phenotype** |  |  |  |  |  |  |
| Low impact, worsening | 2.11 (1.59 - 2.80) | < .001 | 1.36 (1.02 - 1.81) | .04 | 1.01 (0.76 - 1.35) | .92 |
| Moderate impact, worsening | 1.32 (1.01 - 1.74) | .05 | 1.11 (0.86 - 1.44) | .41 | 0.89 (0.69 - 1.15) | .39 |
| High impact, stable | 3.24 (2.49 - 4.20) | < .001 | 2.02 (1.55 - 2.63) | < .001 | 1.14 (0.86 - 1.5) | .37 |
