# Supplementary material for: Complex pain phenotypes: Suicidal ideation and attempt through latent multimorbidity
Source: PLoS One. 2022 Apr 29;17(4):e0267844. doi: 10.1371/journal.pone.0267844 (PMC9053801; doi:10.1371/journal.pone.0267844)
Supplement: S3 Table — (DOCX) [file pone.0267844.s003.docx]

**S3 Table. Multinomial logistic regression by complex pain phenotype for suicidal ideation.**

| **Characteristic** | **No adjustment** | **P value** | **Short set** | **P value** | **Long set** | **P value** |
| --- | --- | --- | --- | --- | --- | --- |
| **Complex pain phenotype** |  |  |  |  |  |  |
| Low impact, worsening | 2.26 (1.66 - 3.09) | < .001 | 1.54 (1.13 - 2.11) | .007 | 1.11 (0.81 - 1.51) | .52 |
| Moderate impact, worsening | 1.37 (1.01 - 1.86) | .04 | 1.22 (0.91 - 1.63) | .18 | 0.95 (0.72 - 1.25) | .70 |
| High impact, stable | 3.23 (2.41 - 4.33) | < .001 | 2.26 (1.69 - 3.03) | < .001 | 1.12 (0.82 - 1.52) | .47 |
