# Supplementary material for: Complex pain phenotypes: Suicidal ideation and attempt through latent multimorbidity
Source: PLoS One. 2022 Apr 29;17(4):e0267844. doi: 10.1371/journal.pone.0267844 (PMC9053801; doi:10.1371/journal.pone.0267844)
Supplement: S4 Table — (DOCX) [file pone.0267844.s004.docx]

**S4 Table. Multinomial logistic regression by complex pain phenotype for suicide attempt.**

| **Characteristic** | **No adjustment** | **P value** | **Short set** | **P value** | **Long set** | **P value** |
| --- | --- | --- | --- | --- | --- | --- |
| **Complex pain phenotype** |  |  |  |  |  |  |
| Low impact, worsening | 1.55 (0.81 - 2.96) | .19 | 0.75 (0.39 - 1.47) | .41 | 0.64 (0.32 - 1.30) | .22 |
| Moderate impact, worsening | 1.13 (0.62 - 2.07) | .70 | 0.75 (0.43 - 1.32) | .32 | 0.67 (0.37 - 1.19) | .17 |
| High impact, stable | 3.25 (1.87 - 5.67) | < .001 | 1.18 (0.65 - 2.15) | .58 | 1.19 (0.66 - 2.16) | .57 |
