# Supplementary material for: Complex pain phenotypes: Suicidal ideation and attempt through latent multimorbidity
Source: PLoS One. 2022 Apr 29;17(4):e0267844. doi: 10.1371/journal.pone.0267844 (PMC9053801; doi:10.1371/journal.pone.0267844)
Supplement: S5 Table — Adjusted odds ratios and 95% confidence intervals associated with pain phenotypes derived from logistic regression analyses: unadjusted, adjusting away confounding associated with sociodemographic, military characteristics, mental health (minus prior suicide-related behavior) covariates using IPSW. (DOCX) [file pone.0267844.s005.docx]

**S5 Table. Binomial logistic regression by complex pain phenotype for suicidal ideation or attempt.** ^a^

| **Characteristic** | **No adjustment** | **P value** | **Long set** | **P value** |
| --- | --- | --- | --- | --- |
| **Complex pain phenotype** |  |  |  |  |
| Low impact, worsening | 1.55 (0.81 - 2.96) | .19 | 1.18 (0.89 - 1.55) | .26 |
| Moderate impact, worsening | 1.13 (0.62 - 2.07) | .70 | 0.96 (0.74 - 1.23) | .73 |
| High impact, stable | 3.25 (1.87 - 5.67) | < .001 | **1.36 (1.04 - 1.77)** | **.02** |

^a^ Adjusted odds ratios and 95% confidence intervals associated with pain phenotypes derived from logistic regression analyses: unadjusted, adjusting away confounding associated with sociodemographic, military characteristics, mental health (minus prior suicide-related behavior) covariates using IPSW
