# Supplementary material for: Complex pain phenotypes: Suicidal ideation and attempt through latent multimorbidity
Source: PLoS One. 2022 Apr 29;17(4):e0267844. doi: 10.1371/journal.pone.0267844 (PMC9053801; doi:10.1371/journal.pone.0267844)
Supplement: S6 Table — Adjusted odds ratios and 95% confidence intervals associated with pain phenotypes derived from logistic regression analyses: unadjusted, adjusting away confounding associated with sociodemographic, military characteristics, mental health (minus prior suicide-related behavior) covariates using IPSW. (DOCX) [file pone.0267844.s006.docx]

**S6 Table. Multinomial logistic regression by complex pain phenotype for suicidal ideation and attempt.** ^a^

| **Characteristic** | **No adjustment** | **P value** | **Suicide ideation** | **P value** | **Suicide attempt** | **P value** |
| --- | --- | --- | --- | --- | --- | --- |
| **Complex pain phenotype** |  |  |  |  |  |  |
| Low impact, worsening | 1.55 (0.81 - 2.96) | .19 | 1.20 (0.89 - 1.63) | .23 | 1.05 (0.54 - 2.05) | .88 |
| Moderate impact, worsening | 1.13 (0.62 - 2.07) | .70 | 0.95 (0.72 - 1.25) | .71 | 0.98 (0.55 - 1.76) | .95 |
| High impact, stable | 3.25 (1.87 - 5.67) | < .001 | 1.25 (0.93 - 1.67) | .14 | **1.87 (1.06 - 3.29)** | **.03** |

^a^ Adjusted odds ratios and 95% confidence intervals associated with pain phenotypes derived from logistic regression analyses: unadjusted, adjusting away confounding associated with sociodemographic, military characteristics, mental health (minus prior suicide-related behavior) covariates using IPSW
