# Supplementary material for: Complex pain phenotypes: Suicidal ideation and attempt through latent multimorbidity
Source: PLoS One. 2022 Apr 29;17(4):e0267844. doi: 10.1371/journal.pone.0267844 (PMC9053801; doi:10.1371/journal.pone.0267844)
Supplement: Attachment — Submitted filename: Response to Reviewers.docx [file pone.0267844.s007.docx]

**Response to reviewers**

**Reviewer #1:** This is an interesting and rare paper focusing on the association of pain and suicidal behavior and specifically if pain phenotypes are independently associated with suicidal ideation or attempts, or via comorbidities, in a longitudinal retrospective cohort study in 9/11 veterans. The objectives are both scientifically and clinically relevant, methods well-chosen and described, results are well presented, thoroughly discussed, limitations are exhaustively addressed and the conclusions are fully supported by the data. As this is a very well designed study and the results are reported in a similarly excellently written paper I only have a few remarks:

1. Please make the title more expressive of the actual major findings of the study. We edited the title per reviewer #1’s recommendations.
2. In the abstract methods section please describe pain phenotypes and also provide some information on the statistical analyses performed. We updated the abstract per reviewer #1’s recommendations.
3. Given the reluctance of psychiatric patients to seek help in part due to stigma but also as a symptom of their psychiatric morbidity, and knowing that the risk of suicidal behavior is higher among untreated psychiatric patients (including PTSD patients), this should be mentioned as a limitation. We updated the limitations and added two references (#53 & 54) to address the reviewer’s comments, see the limitations section, paragraph three, lines 24-28.
4. What was the reason for not including completed suicides as an outcome? At the time the study was completed, we did not have access to data on completed suicides. Thus, our aim was to identify suicidal ideation and suicide attempts that were documented in the medical records.
5. Why were alcohol or substance use disorders not included as covariates? Substance use disorders were included as covariates, see table 1.

**Reviewer #2:** The authors reported original research results derived from their investigation on suicidal ideation and attempt among Post-9/11 Veterans with mild traumatic brain injury and complex pain. The article is overall well-written and of interest to the journal.

1. However, it is unclear how authors assessed suicidal ideation and suicide attempts. Especially for suicidal ideation, the assessment might be more critical. We used ICD9-CM codes used in prior studies of SRB to identify suicide ideation (V6284) and attempt (E950, E952, E953, E953, E954, E955, E956, E957, E958, E959) in national VA inpatient and outpatient data in years 6-8 of VA care.
2. Furthermore, I suggest providing some understanding of the mental pain occurring in the suicidal mind, discussing papers such as Critical appraisal of major depression with suicidal ideation. Ann Gen Psychiatry. 2019. We added a sentence to the introduction, paragraph three, lines 22-24 along with the suggested citation (#18).
